# S‐adenosyl methionine‐AMP opens another skylight of CRISPR system: discovering novel second messenger for bacterial antiviral immunity

**DOI:** 10.1002/mco2.522

**Published:** 2024-04-27

**Authors:** Yongye Huang, Sihui Zhang, Min Wu

**Affiliations:** ^1^ Key Laboratory of Bioresource Research and Development of Liaoning Province College of Life and Health Sciences Northeastern University Shenyang China; ^2^ Wenzhou Traditional Chinese Medicine Hospital of Zhejiang Chinese Medical University Wenzhou China; ^3^ Wenzhou Institute University of Chinese Academy of Sciences Wenzhou Zhejiang China

**Keywords:** antiviral, CRISPR, second messenger

1

In a recent article appeared in *Nature*, Chi et al.^1^ have described S‐adenosyl methionine (SAM)‐AMP as a novel class of second messengers for antiviral signals. They elucidate a previously unknown signaling molecule in bacterial defense systems, opening a new avenue to discover undefined bioactive molecules to expand the repertoire of type III clustered regularly interspaced short palindromic repeats–CRISPR‐associated (CRISPR–Cas) systems.

The old adage “defense wins championships” announces a winning system across all sports. In fact, defense is the basic survival law for all kinds of organisms. In bacteria and archaea, there are multiple sophisticated lines of active defense, called prokaryotic immune system, to stand up to the frequent offense of viruses (bacteriophages).^2^ Numerous antiviral immune systems, including restriction modification (R‐M) and CRISPR–Cas systems,^2^ have been discovered and elucidated in bacteria over the past few years. Bacteria and bacteriophages are in a constant arm race, and messengers play a central role in the offence and defense network.

CRISPR/Cas adaptive immune systems, widespread in prokaryotes, are well‐known bacterial defense systems against foreign mobile genetic elements. There are six types of CRISPR–Cas systems, being divided into two classes: class I systems and class II systems. Class I systems, including type I, type III, and type IV, usually consist of several units of Cas proteins. Class II systems, including type II, type V, and type VI, contain a single effector protein. Among them, type III CRISPR–Cas systems exhibit unique and intriguing characteristics in RNA cleavage and RNA‐activated DNA cleavage for antiviral defense.^3^

Cas10, the signature subunit of type III CRISPR–Cas effectors, harbors a cyclase activity that converts ATP into cyclic oligoadenylates following binding to foreign RNA. In general, type III Cas10 proteins contain an N‐terminal histidine–aspartate nuclease domain, two palm domains, and a C‐terminal domain that anchors Cas10 to other subunits in the respective Csm (type III‐A) or Cmr (type III‐B) complex. Type III‐B CRISPR–Cas systems are of wide interest due to the RNA‐targeting properties but the underlying mechanisms are still obscure.

Cyclic oligonucleotides, a major class of nucleotide second messengers, are involved in the activation of CRISPR, CBASS, Thoeris, and Pycsar defense system.^2^, ^4^ It has been implicated that cyclic oligonucleotides play central role in bacterial antiviral immune defense against invading viruses. Cyclic GMP‐AMP signaling molecules have been found to be common in bacteria as a component of antiphage defense system. In Chi et al.^1^ study, SAM‐AMP is identified as a class of signaling molecule for the first time (Figure 1). It is well known that there is a mutual conversion between AMP and ATP. Cyclic adenosine monophosphate is intracellular second messenger that functions in various physiological processes, and cyclic di‐AMP is the first recognized cyclic oligonucleotides second messengers in bacteria. SAM, commonly known as a trialkyl sulfonium molecule, is widely associated with methyl transfer, acyl carrier protein transfer, and adenosine transfer reactions in multiple physiological metabolic pathways in mammalian cells. In bacteria, SAM is produced to deposit marks on the DNA to help bacteria distinguish their own genome from foreign genetic fragments. The critical finding that SAM‐AMP acts as a class of antiviral second messengers not only broaden our understanding of bacterial molecules represented by SAM, but also further sheds light on the prokaryotic studies in view of evolutionary relationship from eukaryotic signaling pathways.

**FIGURE 1:**
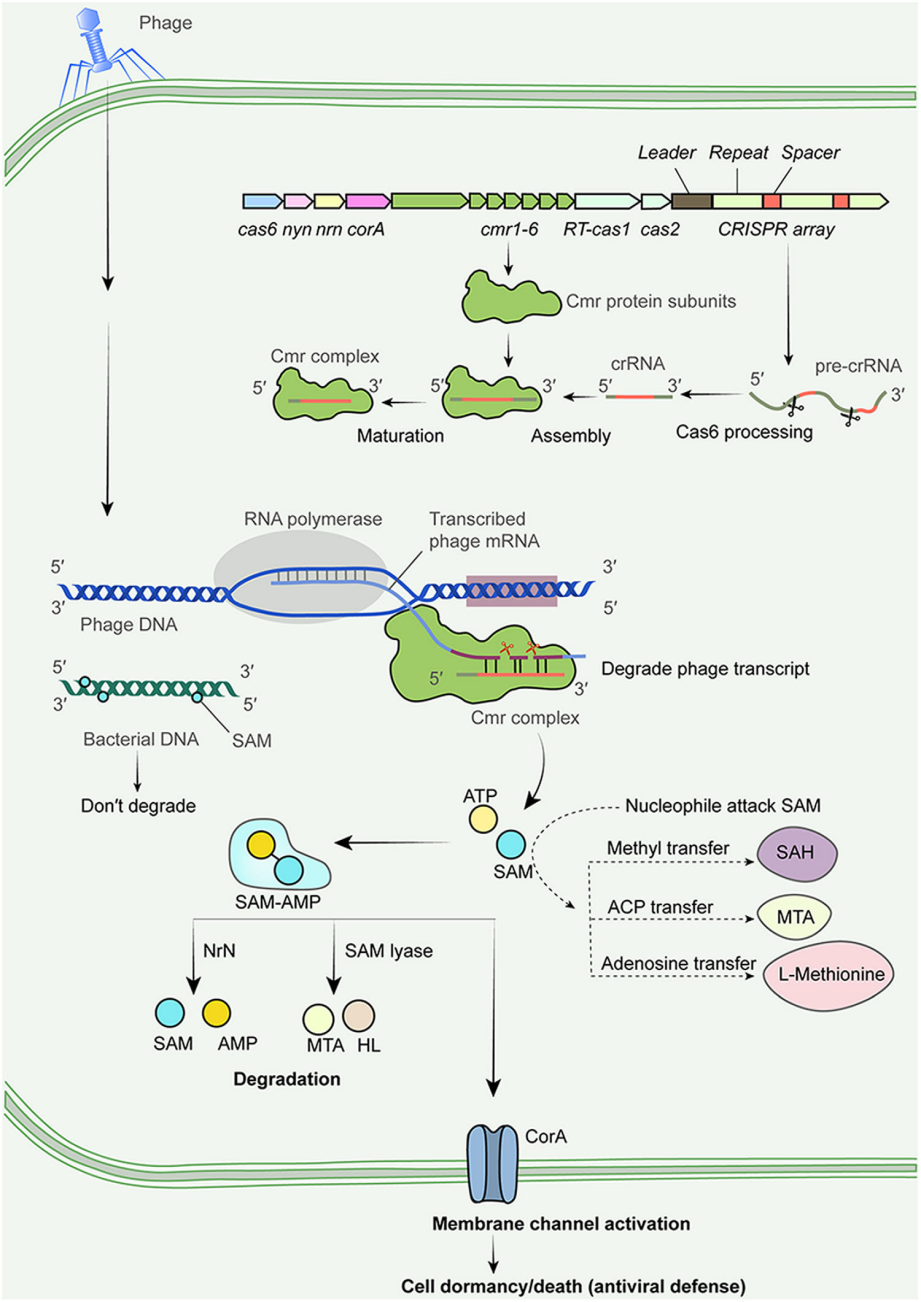
A new class of immune signaling secondary messenger SAM‐AMP has been discovered in *B. fragili*s. In the type III ‐B CRISPR/Cas system‐mediated bacterial defense, CRISPR arrays produce the pre‐crRNA transcripts that are processed by cleavage of Cas6 to form crRNA within a single unit of spacer. CRISPR/Cas gene cassettes yield Cas proteins, including Cas6 and III‐B Cas10 (Cmr) subunits. Subsequently, crRNA together with Cmr subunits are assembled by many steps, including the trimming of the 3′‐ends of crRNA and further forming mature Cmr complex. This complex captures foreign RNA from viral genomic transcription due to the complementarity to crRNA (purple) and exert RNase effect to cleave phage RNA, followed by Cmr complex promoting SAM‐AMP formation.

*Bacteroides fragilis*, an anaerobic organism commonly existing in commensal flora, were selected as the subjects for investigating antiviral signaling associated with type III CRISPR–Cas systems. In *B. fragilis*, the histidine–aspartate nuclease domain is absent in Cas10 that harbors the intact cyclase domain. The authors performed a plasmid challenge assay by expressing wild‐type and cyclase‐defective (Cas10 D328A/D329A variant) Cmr programmed with targeting (pCRISPR‐Tet) or nontargeting (pCRISPR‐pUC) crRNA in *Escherichia coli*. Their results indicate that *B. fragilis* Cmr system is active in vivo depending on the activity of the Cas10 cyclase domain and the presence of effector proteins NrN and CorA.^1^

Based on a authors’ previous finding that activation of the wild‐type Cmr in vitro generated nearly no observable product under incubation with ATP, which prompted the authors to examine whether a vital element of the signaling pathway is missing in the in vitro investigations. The data from high‐performance liquid chromatography and mass spectrometry suggest that there might be uncharacterized cyclin nucleotide or metabolite, and results of tandem mass spectrometry (MS/MS) identify a novel molecule named SAM‐AMP.^1^ By reconstituting the reaction in vitro with ATP and SAM and utilizing HPLC and thin‐layer chromatography for analyzing products, Chi et al.^1^ further verified that *B. fragilis* Cmr could synthesize SAM‐AMP.^1^ Based on the enzyme activity of Cas10 family in synthesizing 3′−5′ phosphodiester bonds and the observation that nuclease P1 could only partially degrade SAM‐AMP, the 3′−5′ phosphodiester linkage is likely responsible for the fusion of SAM to AMP.^1^ Variants of Cas10 with E151R and D70N/E151R mutations show low or even no detectable SAM‐AMP synthase activity, and D70N/E151R mutations exhibit elevated pppApA synthase activity.^1^ These lines of evidence together confirm the existence of SAM‐AMP in *B. fragilis* Cmr system and reveal the enzymology in the conjugate of SAM to AMP.

CorA membrane proteins are virtually ubiquitous and maintain homeostasis in the bacteria. The authors found that CorA is able to bind the SAM‐AMP to implement antiviral function, and variants of CorA with R152–R153 and D219–D220 (within putative SAM‐AMP‐binding site) mutating into alanine exhibit no immunity.^1^ *B. fragilis* NrN protein could specifically degrade SAM‐AMP, providing a type of “off switch” to rebuild antiviral defense systems.^1^ Besides NrN protein, SAM lyase from *C. botulinum* is also found to degrade SAM‐AMP.^1^ Possibly, the SAM‐AMP signaling molecule functions in various types of bacteria. Interestingly, some bacteriophages encode SAM lyase, suggesting the evolution of tit‐for‐tat between bacteria and bacteriophages.

Bacteriophages have also evolved effective mechanisms to overcome the CRISPR–Cas system, such as inducing a point mutation in the PAM. Recently, bacteriophages have been shown to inhibit CRISPR–Cas immunity via a small noncoding RNA anti‐CRISPRs mimicking the repeats in CRISPR.^5^ Therefore, it would be fascinating to determine the mechanisms by which bacteriophages modulate the expression and activity of SAM lyase to destroy the bacterial SAM‐AMP signaling. Studies are also needed to uncover the toxicity of CorA in the situation that there are no synthesized SAM‐AMP and NrN. Cyclic di‐AMP and cyclic di‐GMP are important intracellular nucleotide second messengers that transduce extracellular stimuli to trigger a cascade of cellular responses in bacteria. High intracellular cyclic di‐GMP level relieves environment‐sensing regulator H‐NS‐mediated transcriptional silencing. The selection and activation mechanisms of SAM‐AMP and the other known or unknown second messengers upon environmental cues still need to be explored. The CRISPR–Cas system is an advanced gene editing tool. Engineering molecules, such as SAM‐AMP, are expected to target key domains to generate a CRISPR–Cas system with unique function, including high editing efficiency and low off‐target rates.

The present findings open a skylight of CRISPR–Cas system and would specially be valuable for boosting the research on antiviral immunity. This promising study is expected to be specifically beneficial to maintain the homeostasis of gut flora, accelerate the development of gene editing technologies, and provide a new angle for viewing the biological systems’ relationship of eukaryotes with other systems, such as bacteria.

## AUTHOR CONTRIBUTIONS

Y. Y. H. and M. W. composed and edited the manuscript. S. H. Z. illustrated the figure and artwork in consultation with coauthors. The article has received approval from all authors.

## CONFLICT OF INTEREST STATEMENT

The authors declare no conflict of interest.

## FUNDING INFORMATION

This study was funded by National Natural Science Foundation of China (Nos. 81502582), Fundamental Scientific Research Fund of Liaoning Provincial Education Department (LJKQZ2021002), and Construction Project of Liaoning Provincial Key Laboratory, China (2022JH13/10200026).

## ETHICS STATEMENT

Not applicable.

## Data Availability

Not applicable.
